# Survey Results of a Pilot Sleep Study Near Atlanta International Airport

**DOI:** 10.3390/ijerph16224321

**Published:** 2019-11-06

**Authors:** Sarah Rocha, Michael G. Smith, Maryam Witte, Mathias Basner

**Affiliations:** Unit for Experimental Psychiatry, Division of Sleep and Chronobiology, Department of Psychiatry, University of Pennsylvania Perelman School of Medicine, Philadelphia, PA 19104, USA; rochas@pennmedicine.upenn.edu (S.R.); michael.smith@pennmedicine.upenn.edu (M.G.S.); maryam.witte@duke.edu (M.W.)

**Keywords:** sleep disturbance, pilot field study, aircraft noise, annoyance, postal questionnaire

## Abstract

Aircraft noise can disturb the sleep of residents living near airports. To investigate potential effects of aircraft noise on sleep, recruitment surveys for a pilot field study were mailed to households around Atlanta International Airport. Survey items included questions about sleep quality, sleep disturbance by noise, noise annoyance, coping behaviors, and health. Of 3159 deliverable surveys, 319 were returned (10.1%). Calculated outdoor nighttime aircraft noise (*L*_night_) was significantly associated with lower sleep quality (poor or fair; odds ratio (OR) = 1.04/decibel (dB); *p* < 0.05), trouble falling asleep within 30 min ≥1/week (OR = 1.06/dB; *p* < 0.01), and trouble sleeping due to awakenings ≥1/week (OR = 1.04/dB; *p* < 0.05). *L*_night_ was also associated with increased prevalence of being highly sleep disturbed (OR = 1.15/dB; *p* < 0.0001) and highly annoyed (OR = 1.17/dB; *p* < 0.0001) by aircraft noise. Furthermore *L*_night_ was associated with several coping behaviors. Residents were more likely to report often or always closing their windows (OR = 1.05/dB; *p* < 0.01), consuming alcohol (OR = 1.10/dB; *p* < 0.05), using television (OR = 1.05/dB; *p* < 0.05) and using music (OR = 1.07/dB; *p* < 0.05) as sleep aids. There was no significant relationship between *L*_night_ and self-reported general health or likelihood of self-reported diagnosis of sleep disorders, heart disease, hypertension or diabetes. Evidence of self-reported adverse effects of aircraft noise on sleep found in this pilot study warrant further investigation in larger, more representative subject cohorts.

## 1. Introduction

Postal surveys are a useful tool for investigating attitudes towards environmental noise in communities exposed to aircraft noise [1]. Aircraft noise is often a significant source of annoyance to residents living near airports [1,2]. There has been an indication that the level of annoyance to aircraft noise, which is often paralleled with self-reported sleep disturbance, has been increasing in recent years [3,4]. If attitudes towards aircraft noise amongst affected communities are changing, this may imply a similar shift in sleep disturbance from aircraft noise.

Sleep disturbance is a common concern for residents living near airports. As sleep is a vital process that serves many critical biological functions [5,6,7], disruptions to sleep can have negative behavioral and physiological consequences [8]. When sleep is restricted on a chronic basis, it has been associated with many negative long-term health outcomes, such as increased risk of obesity, heart disease, and diabetes [7]. It is thought that sleep disturbance caused by environmental noise may have similar long-term health implications [9,10]. Because of this, sleep disturbance is regarded as one of the most important non-auditory effects of aircraft noise [11]. However, current literature on how aircraft noise affects sleep for residents near United States (US) airports is lacking. The most recent field studies in the US on aircraft noise-induced sleep disturbance date back to the mid-1990s [12,13]. Over the past several decades, air traffic patterns have changed substantially, with noise levels from single aircraft decreasing but overall traffic volume increasing [2]. The exposure–response relationships for sleep based on previous aircraft noise data may no longer accurately represent current trends in aircraft noise-induced sleep disturbance. Though there have been significant recent field and survey studies in Europe and Asia [14] examining the effects of aircraft noise on sleep, the research methodologies and noise metrics used are often different than those of US field studies, and exposure–response relationships may be influenced by regional differences in sensitivity and attitudinal biases towards noise [15]. More current survey and field studies conducted at multiple airports with different nighttime operation patterns are needed to better understand the effects of aircraft noise on the sleep of residents living near US airports. A nationally representative US field study can provide an update to current noise-exposure response curves and provide information about residents’ attitudes and perceptions of sleep disturbance by aircraft noise. In preparation for this national sleep study (NSS), we performed a pilot study to assess the feasibility of the study methodology, in which we recruited participants for the field study using postal questionnaires to screen for eligible and interested individuals. As only a small subset of respondents will be both interested in and eligible for the field study, we were also interested in whether we could successfully use these recruitment questionnaires to obtain cross-sectional data on associations between aircraft noise and community response. An evaluation of resident responses to the postal recruitment survey against calculated nocturnal aircraft noise levels forms the basis of this paper.

## 2. Methods

This pilot study was performed around Hartsfield–Jackson Atlanta International Airport (ATL). To recruit participants living in the vicinity of the airport, we sent a series of postal surveys. While the primary purpose of the postal survey was to recruit participants into the field study, the survey also gathered data on subjective measures of sleep quality, self-reported disturbance of sleep by noise, coping behaviors in response to nighttime noise, and the overall health of the surveyed population. The study was reviewed and approved by the University of Pennsylvania Institutional Review Board (IRB) (Protocol number 823726). Regarding responses to the postal survey, the IRB reviewed and approved a waiver of written documentation of consent as per US Department of Health and Human Services Title 45—Public Welfare of the Code of Federal Regulations part 46.117 (c) (2).

### 2.1. Survey Methodology

We mailed 3600 surveys to randomly selected households around ATL that had a minimum calculated average nighttime aircraft noise exposure of 35 decibels (dB) (see below for details on how this was calculated). We sent surveys to an equal number of households east and west of ATL (*n* = 1800), and to an equal number of households in five regions that we stratified by noise level in 5 dB increments (*n* = 720 per stratum) to ensure sampling from a wide range of noise exposures. Across 15 mailing waves of equal size (*n* = 240 households per wave), households were sampled from autumn to early summer. One of the overarching aims of this pilot study was to determine a postal survey strategy that would optimize response and recruitment rates for the NSS. As such, survey length, incentive and number of follow-ups were varied over the mailing waves, and analysis of the effectiveness of these different strategies is reported elsewhere [16]. Surveys could be completed online or returned via mail, and the instructions indicated that only a single household member should fill out the survey (the person who most recently celebrated a birthday). The complete survey is provided in the Supplemental Materials (Questionnaire S1), and relevant items for obtaining the required independent variables are discussed below.

### 2.2. Independent Variables: Nighttime Aircraft Noise Exposure

A model of aircraft noise exposure around ATL was needed to analyze the associations between levels of noise exposure and self-reported sleep outcomes and annoyance. We modelled outdoor noise exposure as *L*_night_, which is expressed in decibels (dB) and defined as the A-weighted long-term average sound pressure level between 23:00–07:00. A-weighting is a method of frequency filtering a sound signal to approximate human perception, because human hearing is increasingly insensitive at very low and very high audio frequencies.

**Figure 1:**
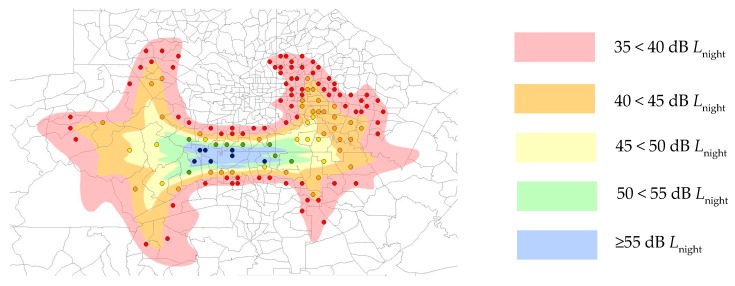
Nighttime aircraft noise contours and census tract boundaries around Hartsfield–Jackson Atlanta International Airport (ATL) airport, modeled using 84 nights of data from 2014–2015. Points indicate the population weighted centroid of each census tract, colored according to the noise contour in which it is located.

Radar data from 2014–2015 were provided for the airport by the US Federal Aviation Administration (FAA). These radar data include flight trajectory, altitude, aircraft type and time information, which were used to as inputs in the FAA’s Integrated Noise Model (INM) to calculate noise levels for each individual aircraft over 84 nights. Noise data during the nighttime period were used to calculate outdoor nighttime noise level (*L*_night_) contours using the INM. We stratified areas into five noise exposure categories: 35 < 40 dB, 40 < 45 dB, 45 < 50 dB, 50 < 55 dB and ≥55 dB *L*_night_, which are shown in Figure 1. 

In the regression modelling (see Statistical Analysis below), *L*_night_ was coded as a continuous independent variable. Results for the association between aircraft noise exposures and the outcome being investigated are, therefore, in terms of odds ratio per 1 dB increase in *L*_night_.

### 2.3. Dependent Variables: Annoyance and Sleep Disturbance by Noise 

Information on the self-reported annoyance and sleep disturbance by aircraft noise over the preceding 12 months were obtained from 5-level Likert scales worded according to recommendations of the International Commission on the Biological Effects of Noise (ICBEN) [17]. Annoyance and sleep disturbance were each recoded into dichotomous variables by assigning the three lowest response levels (“not at all” to “moderately”) a value of 0 and the two highest response levels (“very” or “extremely”) a value of 1.

### 2.4. Dependent Variables: Sleep Quality 

The auditory system remains active during sleep, and noise can interfere with subjective sleep [18]. We obtained information on habitual sleep quality over the past month using 4-level Likert-type items from the Pittsburgh Sleep Quality Index [19]. Overall sleep quality was coded as a dichotomous variable by assigning the two highest response levels (“fairly good” and “very good”) a value of 0 and the two lowest response levels (“very bad” and “fairly bad”) a value of 1. Trouble falling asleep, nocturnal awakenings, sleep medication use, and daytime sleepiness were recoded as dichotomous variables by assigning the two lowest response levels (“not during the past month” and “less than once a week”) a value of 0 and the two highest response levels (“once or twice a week” and “three or more times a week”) a value of 1.

### 2.5. Dependent Variables: Coping Behaviors 

If an individual feels that noise is interfering with their activity, including sleep, they may consequently adopt noise-mitigating behaviors. We therefore obtained use of sleep aids due to environmental noise when trying to sleep from the survey. Respondents estimated how often over the past month they “wear earplugs,” “use alcohol,” “use medication,” “turn on the TV,” “turn on music,” “close windows,” “use a sound machine,” or “turn on a fan” on a 5-level Likert-type scale. Each coping behavior was recoded into dichotomous variables by assigning the three lowest response levels (“never” to “sometimes”) a value of 0 and the two highest response levels (“often” or “always”) a value of 1.

### 2.6. Dependent Variables: Health

There is some evidence that chronic noise exposure is associated with adverse health outcomes [18]. Respondents rated their general health on a 5-level Likert-type scale. We recoded general health into a dichotomous variable by assigning the three highest ratings (“good”, “very good” and “excellent”) a value of 0 and the two lowest ratings (“poor” and “fair”) a value of 1.

Respondents indicated (yes/no) any previous diagnosis of hypertension, migraines, arrhythmia, heart disease, stomach ulcer, or diabetes. Each of these conditions was coded as a dichotomous variable, where any positive indication of a diagnosis was scored as 1, and no indication of a diagnosis was scored as 0.

Respondents indicated (yes/no) any previous diagnosis of sleep apnea, periodic limb movement syndrome, narcolepsy, insomnia, restless leg syndrome, or another sleep disorder. Sleep disorder was coded as a dichotomous variable, where any positive indication of a diagnosis of one or more sleep disorders was scored as 1, and no indication of a diagnosis was scored as 0.

### 2.7. Individual-Level Covariates

Noise sensitivity may influence subjective responses to environmental aircraft noise [20]. Noise sensitivity items in the survey were taken from the Weinstein Noise Sensitivity Scale [21] and rated on a 6-point numerical scale with endpoints of 1 (“Strongly disagree”) and 6 (“Strongly agree”). We coded noise sensitivity as a dichotomous variable (noise-sensitive/not sensitive) according to responses to the single item “I am sensitive to noise.” We classified a respondent as noise-sensitive if they gave a response of 5 or 6, and as not sensitive if they gave a response of 1 to 4.

Individuals with hearing problems may perceive aircraft noise less well than individuals without hearing problems, and could therefore be less susceptible to potential noise effects. We collected dichotomous (yes/no) information on problems or difficulties with hearing from a single survey item. 

We obtained the following survey data on individual-level variables that could influence sleep: sex (female/male), age (continuous), body mass index (BMI, continuous), race, ethnicity, marital status (categorical), and socioeconomic factors: household income bracket (ordinal), occupational status (categorical) and education (categorical). We collapsed income into a 4-level ordinal variable (<$50,000; $50,000–$100,000; >$100,000; Prefer not to answer). 

Demographic data for census tracts from which we sampled (Figure 1) were extracted from American FactFinder (http://factfinder2.census.gov/), and are given in Supplemental Table S1.

### 2.8. Statistical Analysis

We analyzed data in logistic regression models using SAS (version 9.4, SAS Institute, Cary, NC, USA). We first analyzed each outcome separately in a crude, unadjusted model, with *L*_night_ only as an independent variable. We then analyzed each outcome in an adjusted multilevel regression model. We used directed acyclic graphs in DAGitty v2.3 to determine the minimal adjustment required to estimate the total effect of *L*_night_ on outcomes of interest (see Supplemental Figures S1–S3) [22]. Adjustments for age and income were minimally necessary, so we did not include occupational status or education in analysis models. In addition to *L*_night_ and income, we furthermore included sex, BMI, noise sensitivity and hearing problems as covariates in the adjusted model since we were interested in their influence on our outcomes. Fifteen missing values for age and 14 missing values for BMI were replaced with the mean age (53 years) and mean BMI (29 kg/m^2^). Where categorical covariate data (sex, income, hearing problems and/or noise sensitivity) were missing, we excluded the respondent from analysis.

Wald Chi-Squared tests were performed to determine the significance of the predictor variables, and statistical significance was set to α = 0.05. We did not correct for multiple testing in this exploratory analysis of pilot study data. Odds ratios (OR) with 95% confidence intervals (CI) and *p*-values are reported both for unadjusted and adjusted models.

## 3. Results

Out of the 3600 surveys mailed, 3159 surveys could be delivered. Of the deliverable surveys, 319 were completed and returned, resulting in a response rate of 10.1%. Twenty one surveys with missing information for sex (*n* = 21), income (*n* = 9), noise sensitivity (*n* = 7) and/or hearing problems (*n* = 33) were excluded from analysis, resulting in an effective sample size of *n* = 268 (8.5%) of the surveyed population. The number of responses in each dichotomous outcome category are given in Supplemental Tables S2–S4. 

### 3.1. Population Demographics

Table 1 summarizes the demographics for respondents to the noise and sleep survey for whom there were no missing sex, income, noise sensitivity or hearing problem data. There were 57 participants in the 35 < 40 dB noise exposure category, 46 in the 40 < 45 dB category, 51 in the 45 < 50 dB category, 64 in the 50 < 55 dB category and 50 in the ≥55 dB category. Respondents ranged in age from 21 to 97 years, with a mean of 52.4 years (standard deviation (SD) ± 15.2) and had a mean BMI of 29.3 kg/m^2^ (SD ± 6.5). Sixty one percent of respondents were black, which is a similar proportion to the 62.5% mean proportion for the sampled sampling region (see in Supplemental Table S1). For the highest level of completed education, 46.4% of respondents had no college education, which is slightly higher than the 40.0% of the population without college education in the sampling region. Among respondents who disclosed their income, 50.9% had a household income below $50,000, with 31.3% of respondents and the median value lying in the $25–50 k category. This is in agreement with the mean household income for the sampled census tracts of $49,100.

### 3.2. Survey Responses

#### 3.2.1. Sleep Disturbance by Noise, Annoyance by Noise and Sleep Quality

Results of the unadjusted logistic regression models for annoyance, sleep disturbance and sleep quality are presented in Table 2. Exact *p*-values for all tests are given in Supplemental Table S5. With increasing nocturnal aircraft noise exposure, *L*_night_, there were significant increases in the following outcomes: sleep disturbance by aircraft noise, annoyance by aircraft noise, likelihood of rating overall sleep quality as “bad” or “fairly bad”, trouble falling asleep within 30 min at least once a week, trouble sleeping at night due to nocturnal awakenings or waking too early in the morning at least once a week, and trouble staying awake during the daytime at least once a week. Only use of sleep medications was not significantly associated with *L*_night_. Nighttime aircraft noise was therefore associated with higher sleep disturbance and decreased subjective sleep quality.

The odds ratios for the associations between *L*_night_ and sleep in the adjusted regression models (Table 3) closely match the results from the unadjusted models, although trouble staying awake during the daytime is no longer significant. Furthermore, there were significant effects of noise sensitivity for all of the sleep outcomes, with noise-sensitive individuals reporting higher disturbance, annoyance and trouble sleeping than non-sensitive individuals. Respondents with hearing problems were more likely to report trouble falling asleep and staying asleep. There were also effects of income bracket, with respondents in the highest annual income bracket (>$100 k) less annoyed and sleep disturbed by aircraft noise than respondents in the lowest income bracket (<$50 k).

#### 3.2.2. Use of Sleep Aids in Response to Noise

Results of the unadjusted logistic regression models for often or always using different sleep aids are presented in Table 4. Exact *p*-values for all tests are given in Supplemental Table S5. With increasing nocturnal aircraft noise exposure, *L*_night_, respondents were significantly more likely to report using alcohol, television, music and closing their windows in response to noise. Nighttime aircraft noise was therefore positively associated with increased prevalence of a number of coping behaviors.

The odds ratios and tests of significance for the associations between *L*_night_ and sleep aid use in the adjusted regression models (Table 5) closely match the results from the unadjusted models. Furthermore, there were some significant effects of age, hearing problems and noise sensitivity. Older individuals were increasingly less likely to use music or fans as a sleep aid. Noise-sensitive respondents and respondents with hearing problems were more than twice as likely to use either medication or television as a sleep aid. Noise-sensitive individuals were also more likely to close their windows and use fans than non-sensitive individuals. Individuals with hearing problems were over 5 times as likely to use music as a sleep aid against noise compared to individuals without hearing problems.

#### 3.2.3. Health

Results of the unadjusted logistic regression models for self-reported general heath and diagnosis of relevant health outcomes are presented in Table 6. Exact *p*-values for all tests are given in Supplemental Table S5. With increasing nocturnal aircraft noise exposure, *L*_night_, respondents were significantly more likely to rate their health as worse, i.e., as fair or poor rather than good to excellent. This association was not statistically significant after adjusting for BMI, sex, age, hearing problems, noise sensitivity and income (Table 7). With increasing BMI, respondents were more likely to rate their health as worse and report a prior diagnosis of a sleep disorder, hypertension and diabetes. With increasing age, respondents were more likely to report a prior diagnosis of a sleep disorder, hypertension, arrhythmia, heart disease and diabetes. There were no significant effects of sex or noise sensitivity on any of the measured health outcomes. 

## 4. Discussion

The primary purpose of this pilot study was to establish the feasibility of using postal questionnaires to recruit participants into a NSS investigating the relationship between aircraft noise and physiologic sleep. As part of this feasibility assessment, we were interested in the appropriateness of using these survey instruments to capture self-reported associations between aircraft noise and sleep and health in the NSS. To this end, we sent out 3600 postal surveys to residents near ATL that asked questions on self-reported sleep disturbance to noise and annoyance to noise. We also obtained demographic data, self-reported overall sleep quality and noise sensitivity, use of sleep aids in response to noise, and self-reported health information. These surveys allowed for an evaluation of subjective response to aircraft noise in the community, how residents perceive noise has affected their sleep, and how they adapt to noise using coping mechanisms. The results showed that nighttime aircraft noise was associated with sleep disturbance and annoyance, worse subjective sleep, and the adoption of sleep aids to mitigate the effects of noise on sleep.

We found that residents living in regions with higher levels of nighttime aircraft noise were more likely to report poor overall sleep quality. They also reported greater difficulty falling asleep within 30 min and trouble sleeping at night due to waking in the middle of the night or too early in the morning. These findings are consistent with those of a recent World Health Organization (WHO) review on environmental noise and self-reported sleep outcomes [14]. While the WHO report found a statistically significant relationship between aircraft noise and disruptions to sleep only when noise was referred to in the question, in our study, *L*_night_ was associated with poorer self-reported sleep quality without a reference to noise. However, the title of our survey referenced noise, which may have influenced respondents when answering questions about their sleep. Furthermore, the choice of classification we used for coding the dichotomous variables of reporting difficulty sleeping, i.e., we coded a sleep difficulty as present if it occurred once a week or more, rather than the single highest response of three time a week or more, should moderate conclusions regarding associations between aircraft noise and subjective sleep. According to the American Psychiatric Association, a criterion for diagnosis of insomnia is that sleep difficulty occurs at least three times per week [23]. Few respondents reported that trouble with sleep occurred at least three times a week, precluding statistical analysis of this response category only, and further illustrating the need for the larger sample sizes that will be obtained in the NSS. Respondents were increasingly likely to report that they were very or extremely annoyed and sleep disturbed with increasing *L*_night_, which are responses corresponding to the “highly annoyed” and “highly sleep disturbed” classifications used by the WHO in their estimations of the disease burden of environmental noise [11]. These level-dependent associations between aircraft noise and annoyance and sleep disturbance are consistent with what has been found previously in the literature [14,24,25]. Good quality sleep is important for many biological functions and overall health, and so public perception that aircraft noise is disrupting sleep is a relevant concern. However, questionnaires on self-reported sleep may not fully capture the magnitude of the effect of environmental noise on sleep. Nighttime awakenings due to noise often occur without conscious awareness, and so residents may not accurately estimate the degree to which noise affects their sleep. There is also some evidence that self-reported sleep may only be weakly associated with objective sleep measures of sleep [26]. Thus, future field studies on the physiological responses to nighttime aircraft noise are needed to elucidate the objective impact of aircraft noise on sleep.

Along with disrupting sleep, aircraft noise can be annoying to residents living near airports. It was found that residents living in regions with higher *L*_night_ levels were significantly more likely to report feeling highly annoyed by aircraft noise over the last twelve months. This finding is consistent with previous annoyance studies (e.g., [4,27,28]). A limitation of this finding is that we only examined the associations between *L*_night_ and annoyance to aircraft noise, and so we cannot exclude daytime noise exposure as the main source of annoyance. A high level of annoyance to aircraft noise is concerning not just because of its impact on mood, but also because of its potential to influence sleep. While we sleep, the brain continues to processes and evaluate auditory stimuli, and so noise events that have emotional relevancy may induce a nighttime arousal with a higher probability compared to those that are less emotionally relevant [29]. Addressing annoyance to aircraft noise may thus be an important component in preventing aircraft noise-induced sleep disturbance. Because of the limited sample size and low response rate, annoyance levels found in this study should not be generalized to the studied, or other, airports. A much larger national survey across a more representative selection of 20 airports was recently conducted by the FAA and is expected to provide more precise exposure–response functions for daytime and nighttime aircraft noise annoyance [30]. 

Residents who were sensitive to noise were more likely to report annoyance and sleep disturbance by aircraft noise, as well as worse subjective sleep overall in all measures of sleep quality. This is in line with previous findings that noise-sensitive individuals report worse sleep [31,32,33]. As a result of noise, sensitive respondents were also more likely to report using three of the eight surveyed sleep aids at least once a week, further supporting the idea they were more psychologically susceptible to, or more cognizant of, nocturnal noise. It is, however, unclear how sensitivity might influence the impact of noise on sleep biology, with several studies finding minimal or no physiologic effects of sensitivity [32,34,35], or even that their sleep was objectively better [36]. Regardless of whether or not physiologic effects of noise are moderated by an individual’s sensitivity, consistent findings, both here and previously, that they report worse subjective sleep and increased annoyance and disturbance remain relevant when considering the public health implications of nocturnal aircraft noise exposure. 

Those exposed to high levels of aircraft noise during sleep may try to adapt to the noise using various sleeping aids. We found that *L*_night_ was significantly associated with an increased likelihood to frequently close windows when trying to sleep and to use alcohol, television and/or music as sleep aids because of noise. These findings suggest that residents in communities with higher *L*_night_ are concerned with noise affecting their sleep, and they engage in coping behaviors to adapt to the noise at night. A limitation is that our survey questions on sleep aids referenced noise in general, rather than aircraft noise specifically. It may be possible that residents living in neighborhoods with higher *L*_night_ use sleep aids to block out other sources of nighttime noise as well. However, given that these residents were significantly annoyed and disturbed in their sleep by aircraft noise, it is plausible that aircraft noise was the primary noise source that induced these coping behaviors. 

In the long-term, exposure to high levels of aircraft noise may have adverse health consequences [2,18]. It is thought that nighttime aircraft noise exposure increases the risk of cardiovascular disease [10] and is known to disturb sleep, which, when restricted on a chronic basis, is associated with increased risk of cancer, obesity, and diabetes [7]. However, we did not find an association between *L*_night_ and poorer self-reported general health after adjusting for individual-level covariates and sociodemographic factors. Nor did we find an association between *L*_night_ and diagnosis of heart disease, hypertension or diabetes. However, we were underpowered to detect the small effect sizes expected for these health outcomes. The significant relationships between BMI and age with a number of the health outcomes are all positive, as would be expected for sleep disorders, hypertension, heart disease and diabetes, indicating that the questionnaire items may be suitable for capturing the prevalence of diagnosis among the sampled population. 

### Limitations

Our findings have several limitations, most notably that our study population was small, with an effective sample size of 268 (8.5% of the surveyed population). Our response rate was lower (10.1%) than the 46%–76% response rates seen in other postal questionnaires on attitudes towards aircraft noise [37], and survey responses may not accurately represent the attitudes and sleep patterns of the population around ATL. However, this survey was primarily aimed at recruitment for a field study, and a number of the survey rounds used mailing strategies that were ineffective at eliciting responses. A comprehensive analysis of the survey response rate has been completed and is reported in another publication [16], and a higher response rate is expected in the NSS. Despite the low response rate, respondent demographics were similar to the demographics of the census blocks from which we sampled, suggesting a degree of representativeness of the obtained survey data, although we have an overrepresentation of women in our sample.

We did not have information on noise exposure levels in the bedrooms of survey participants. Our survey study used estimated outdoor nighttime aircraft exposure levels based on flight traffic data; however, these estimates may not always reflect actual noise levels in the bedroom. If residents close their windows at night, as was more likely among residents in the most exposed areas, noise levels can be diminished by up to approximately 28 dB [38]. Aircraft noise can also be masked by, for example, noise from air conditioning, television or white noise machines. Accurate bedroom noise levels can only be obtained with measurement, which was outside of the scope of this survey analysis. 

Lastly, because of the exploratory design of this study, we decided not to correct for multiple hypothesis testing, and therefore inferences drawn from these tests may not be reproducible [39]. 

## 5. Conclusions

In this pilot study, average nighttime aircraft noise was significantly associated with self-reports of worse overall sleep quality, trouble falling asleep within 30 min, greater difficulty to maintain sleep, annoyance, and sleep disturbance. Residents in areas exposed to higher levels of aircraft noise coped by closing the windows at night, consuming alcohol, and using television and music as sleep aids. After adjustment for sociodemographic factors, we did not find a significant effect of nocturnal aircraft noise exposure on any of the investigated self-reported health outcomes. Despite the limitations of this pilot study, this cross-sectional study suggests evidence of adverse effects of aircraft noise, warranting further investigation in larger subject cohorts. The results indicate that postal surveys with the primary objective of recruitment into the future NSS can also be used to obtain quality data on annoyance, sleep, health and coping behaviors among populations living around airports.

## Figures and Tables

**Table 1:** Demographic characteristics of survey respondents (*N* = 268) for whom complete data were available for regression analysis. Respondents could provide multiple answers for Race.

Variable	Level	Percent
Sex (*n* = 268)	Women	64.9
	Men	35.1
Race (*n* = 268)	Black	61.2
	White	24.6
	Other	8.2
	Prefer not to answer	10.4
Marital Status (*n* = 267)	Single	36.9
	Married or domestic partners	38.6
	Widowed	7.9
	Separated/divorced	16.5
Income (*n* = 268)	<$50,000	41.8
	$50,000–$100,000	27.2
	>$100,000	13.1
	Prefer not to answer	17.9
Education (*n* = 265)	<High School	4.2
	High School	42.3
	College or greater	53.6
Employment (*n* = 265)	Working	53.6
	Unemployed	9.1
	Student	1.9
	Retired	30.9
	Homemaker	4.5
Hearing (*n* = 268)	No problems	85.8
	Problems	14.2
Noise sensitivity (*n* = 268)	Not sensitive	69.0
	Sensitive	31.0

**Table 2:** Odds ratios and 95% confidence intervals from unadjusted logistic regression models for sleep quality variables.

Covariate	Outcome Measure						
	Sleep Disturbance	Annoyance	Overall Sleep Quality	Trouble Falling Asleep	Trouble Sleeping at Night	Sleep Medication	Trouble Staying Awake
*L*_night_	1.15 (1.10–1.20) ****	1.17 (1.11–1.22) ****	1.05 (1.01–1.08) *	1.05 (1.02–1.09) **	1.04 (1.01–1.08) *	0.99 (0.95–1.04)	1.06 (1.01–1.11) *

*p*-values for odds ratios that are statistically significant are denoted with asterisks (* <0.05; ** <0.01; **** <0.0001).

**Table 3:** Odds ratios (95% confidence intervals) from logistic regression models for sleep quality variables, adjusted for age, body mass index (BMI), sex, hearing problems, noise sensitivity and income.

Covariate	Level	Outcome Measure						
		Sleep Disturbance	Annoyance	Overall Sleep Quality	Trouble Falling Asleep	Trouble Sleeping at Night	Sleep Medication	Trouble Staying Awake
*L*_night_	Continuous	1.15 (1.10–1.21) ****	1.17 (1.11–1.23) ****	1.04 (1.00–1.08) *	1.06 (1.02–1.10) **	1.04 (1.00–1.08) *	0.98 (0.94–1.03)	1.05 (1.00–1.11)
BMI	Continuous	0.95 (0.90-0.99) *	0.95 (0.90–1.00) *	1.00 (0.96–1.04)	0.99 (0.95–1.03)	1.00 (0.95–1.04)	1.04 (0.99–1.09)	1.00 (0.96–1.05)
Sex	Female (ref.)	-	-	-	-	-	-	-
	Male	1.05 (0.54–2.03)	1.13 (0.57–2.24)	0.99 (0.56–1.75)	1.23 (0.71–2.14)	0.59 (0.34–1.05)	0.66 (0.31–1.37)	0.58 (0.31–1.07)
Age	Continuous	1.00 (0.98–1.02)	1.00 (0.98–1.02)	0.99 (0.98–1.01)	0.99 (0.97–1.01)	0.99 (0.97–1.00)	0.99 (0.97–1.02)	0.99 (0.97–1.00)
Hearing problems	No hearing problems (ref.)	-	-	-	-	-	-	-
	Hearing problems	0.72 (0.28–1.85)	0.66 (0.25–1.77)	1.44 (0.68–3.06)	2.46 (1.10–5.51) *	2.51 (1.02–6.15) *	1.57 (0.64–3.85)	1.98 (0.91–4.29)
Noise sensitivity	Not noise-sensitive (ref.)	-	-	-	-	-	-	-
	Noise-sensitive	3.05 (1.59–5.83) ***	3.10 (1.59–6.07) ***	2.09 (1.20–3.66) **	2.74 (1.53–4.89) ***	4.01 (2.05–7.86) ****	2.10 (1.07–4.14) *	2.03 (1.13–3.67) *
Income	<$50 k (ref.)	-	-	-	-	-	-	-
	$50–100 k	0.49 (0.23–1.04)	0.69 (0.32–1.47)	1.08 (0.57–2.07)	0.94 (0.50–1.78)	0.89 (0.45–1.75)	1.13 (0.48–2.67)	1.53 (0.78–2.98)
	>$100 k	0.21 (0.05–0.80) *	0.17 (0.04–0.85) *	0.72 (0.28–1.84)	0.63 (0.27–1.44)	0.72 (0.30–1.71)	1.7 (0.60–4.86)	0.83 (0.31–2.23)
	Prefer not to answer	0.67 (0.29–1.50)	0.85 (0.37–1.96)	0.82 (0.38–1.75)	0.61 (0.29–1.28)	0.56 (0.26–1.21)	1.88 (0.76–4.62)	0.62 (0.27–1.44)

ref. denotes reference category; *p*-values for odds ratios that are statistically significant are denoted with asterisks (* <0.05; ** <0.01; *** <0.001; **** <0.0001).

**Table 4:** Odds ratios and 95% confidence intervals from unadjusted logistic regression models for always or often using sleep aids because of noise.

Covariate	Outcome Measure							
	Earplugs	Alcohol	Medication	TV	Music	Close Windows	Sound Machine	Fan
*L*_night_	1.04(0.98–1.12)	1.11 (1.01–1.21) *	1.01 (0.97–1.06)	1.06 (1.02–1.10) **	1.08 (1.02–1.13) **	1.05 (1.01–1.08) **	0.97 (0.90–1.05)	1.02 (0.99–1.06)

*p*-values for odds ratios that are statistically significant are denoted with asterisks (* <0.05; ** <0.01).

**Table 5:** Odds ratios (95% confidence intervals) from logistic regression models for always or often using sleep aids because of noise, adjusted for age, BMI, sex, hearing problems, noise sensitivity and income.

Covariate	Level	Outcome Measure							
		Earplugs	Alcohol	Medication	TV	Music	Close Windows	Sound Machine	Fan
*L*_night_	Continuous	1.04 (0.96–1.12)	1.10 (1.00–1.21) *	1.01 (0.96–1.06)	1.05 (1.01–1.10) *	1.07 (1.01–1.13) *	1.05 (1.01–1.09) **	0.99 (0.91–1.07)	1.01 (0.97–1.06)
BMI	Continuous	1.08 (1.01–1.15) *	0.99 (0.90–1.09)	1.03 (0.97–1.09)	1.02 (0.96–1.07)	1.04 (0.99–1.09)	0.96 (0.93–1.00)	0.95 (0.85–1.06)	1.03 (0.99–1.08)
Sex	Female (ref.)	-	-	-	-	-	-	-	-
	Male	0.90 (0.30–2.75)	1.12 (0.30–4.12)	0.85 (0.38–1.87)	0.82 (0.38–1.80)	0.84 (0.37–1.91)	1.16 (0.67–1.99)	0.97 (0.30–3.19)	0.77 (0.42–1.43)
Age	Continuous	1.00 (0.96–1.04)	0.97 (0.93–1.01)	1.00 (0.97–1.02)	0.98 (0.95–1.00)	0.94 (0.92–0.97) ****	0.99 (0.97–1.00)	1.00 (0.97–1.0)	0.97 (0.95–0.99) **
Hearing problems	No hearing problems (ref.)	-	-	-	-	-	-	-	-
	Hearing problems	3.00 (0.92–9.76)	0.55 (0.06–4.83)	2.91 (1.22–6.98) *	5.14 (2.13–12.41) ***	5.18 (2.02–13.27) ***	1.05 (0.50–2.22)	1.14 (0.23–5.67)	2.07 (0.95–4.5)
Noise sensitivity	Not noise-sensitive (ref.)	-	-	-	-	-	-	-	-
	Noise-sensitive	2.42 (0.84–6.97)	2.29 (0.67–7.84)	2.29 (1.09–4.81) *	2.37 (1.13–4.97) *	1.08 (0.46–2.52)	1.71 (0.99–2.96)	0.96 (0.28–3.26)	2.04 (1.12–3.71) *
Income	<$50 k (ref.)	-	-	-	-	-	-	-	-
	$50–100 k	2.46 (0.73–8.30)	1.23 (0.25–5.98)	0.89 (0.34–2.30)	1.42 (0.57–3.52)	1.35 (0.55–3.29)	0.98 (0.53–1.83)	1.59 (0.38–6.71)	0.80 (0.39–1.63)
	>$100 k	1.77 (0.30–10.6)	1.12 (0.10–12.12)	1.74 (0.57–5.34)	0.91 (0.22–3.64)	0.45 (0.09–2.28)	0.62 (0.26–1.47)	2.25 (0.43–11.65)	1.00 (0.39–2.54)
	Prefer not to answer	0.91 (0.16–5.15)	1.80 (0.40–8.16)	1.22 (0.44–3.39)	2.51 (0.97–6.49)	0.99 (0.34–2.93)	0.50 (0.24–1.05)	1.13 (0.19–6.61)	0.91 (0.40–2.03)

ref. denotes reference category; *p*-values for odds ratios that are statistically significant are denoted with asterisks (* <0.05; ** <0.01; *** <0.001; **** <0.0001).

**Table 6:** Odds ratios and 95% confidence intervals from unadjusted logistic regression models for general health and diagnosis of different health outcomes.

Covariate	Outcome Measure							
	General Health ^†^	Sleep Disorder	Hypertension	Chronic Headaches/Migraine	Arrhythmia	Heart Disease	Stomach Ulcer	Diabetes
*L*_night_	1.06 (1.02–1.11) **	1.00 (0.96–1.04)	1.00 (0.97–1.04)	1.04 (0.98–1.11)	0.98 (0.92–1.04)	1.06 (0.97–1.15)	0.95 (0.86–1.05)	0.98 (0.93–1.03)

^†^ Odds ratio of reporting health as poor or fair. *p*-values for odds ratios that are statistically significant are denoted with asterisks (** <0.01).

**Table 7:** Odds ratios (95% confidence intervals) from logistic regression models for general health and diagnosis of different health outcomes, adjusted for age, BMI, sex, hearing problems, noise sensitivity and income.

Covariate	Level	Outcome Measure							
		General Health ^†^	Sleep Disorder	Hypertension	Chronic Headaches/Migraine	Arrhythmia	Heart Disease	Stomach Ulcer	Diabetes
*L*_night_	Continuous	1.04 (1.00–1.09)	0.99 (0.95–1.03)	1.00 (0.96–1.04)	1.03 (0.96–1.10)	0.99 (0.92–1.06)	1.08 (0.98–1.18)	0.95 (0.85–1.06)	0.96 (0.90–1.01)
BMI	Continuous	1.08 (1.03–1.13) ***	1.07 (1.02–1.12) **	1.13 (1.07–1.19) ****	0.98 (0.91–1.06)	1.01 (0.93–1.09)	1.02 (0.94–1.11)	0.95 (0.83–1.09)	1.10 (1.04–1.17) ***
Sex	Female (ref.)	-	-	-	-	-	-	-	-
	Male	1.33 (0.68–2.59)	0.85 (0.44–1.62)	1.04 (0.55–1.95)	0.51 (0.16–1.63)	1.14 (0.39–3.31)	1.86 (0.51–6.79)	0.52 (0.09–2.85)	0.82 (0.34–1.97)
Age	Continuous	0.99 (0.97–1.02)	1.03 (1.01–1.05) *	1.10 (1.07–1.13) ****	0.98 (0.95–1.01)	1.07 (1.02–1.11) **	1.06 (1.01–1.12) *	1.03 (0.98–1.08)	1.06 (1.03–1.10) ***
Hearing problems	No hearing problems (ref.)	-	-	-	-	-	-	-	-
	Hearing problems	2.28 (1.00–5.18) *	2.03 (0.93–4.42)	1.25 (0.54–2.90)	1.24 (0.33–4.68)	2.12 (0.67–6.73)	2.27 (0.55–9.25)	0.67 (0.07–6.28)	0.85 (0.28–2.58)
Noise sensitivity	Not noise-sensitive (ref.)	-	-	-	-	-	-	-	-
	Noise-sensitive	1.28 (0.66–2.50)	1.61 (0.86–3.00)	0.87 (0.46–1.65)	1.36 (0.50–3.73)	1.65 (0.60–4.53)	1.02 (0.27–3.82)	0.35 (0.04–2.92)	1.31 (0.58–2.98)
Income	<$50 k (ref.)	-	-	-	-	-	-	-	-
	$50–100 k	0.78 (0.37–1.67)	1.13 (0.53–2.41)	1.15 (0.56–2.37)	0.84 (0.27–2.62)	1.27 (0.39–4.13)	1.42 (0.34–5.94)	0.78 (0.13–4.64)	0.64 (0.22–1.92)
	>$100 k	0.22 (0.05–1.04)	2.03 (0.79–5.26)	1.98 (0.75–5.23)	0.36 (0.04–3.13)	0.94 (0.17–5.34)	1.03 (0.10–10.79)	1.47 (0.21–10.10)	1.3 (0.35–4.83)
	Prefer not to answer	1.30 (0.57–2.96)	1.60 (0.69–3.73)	1.10 (0.48–2.55)	0.60 (0.15–2.39)	0.73 (0.14–3.79)	0.57 (0.06–5.22)	-	3.09 (1.13–8.48) *

^†^ Odds ratio of reporting health as poor or fair; ref. denotes reference category; *p* values for odds ratios that are statistically significant are denoted with asterisks (* <0.05; ** <0.01; *** <0.001; **** <0.0001). Among respondents who chose not to report income, none reported stomach ulcers, so the odds ratio could not be determined.

## Supplementary Materials

The following are available online at https://www.mdpi.com/1660-4601/16/22/4321/s1, Questionnaire S1: Complete postal survey, Table S1: Demographic characteristics of census tracts within each noise category, Table S2: Respondents (n) indicating being disturbed and annoyed by aircraft noise, fair or poor sleep quality, and trouble sleeping due to noise ≥1/week, Table S3: Respondents (n) indicating using sleep aids because of noise often or more frequently, Table S4: Respondents (n) indicating poor or fair general health, diagnosis of any sleep disorder, and diagnosis of relevant health outcomes, Table S5: *p*-values for covariates included in the crude (*L*_night_ only) and fully adjusted regression model for each questionnaire outcome measure, Figure S1: DAG for annoyance/sleep disturbance, Figure S2: DAG for health outcomes, Figure S3: DAG for use of sleep aids.
