# Supplementary material for: Survey Results of a Pilot Sleep Study Near Atlanta International Airport
Source: Int J Environ Res Public Health. 2019 Nov 6;16(22):4321. doi: 10.3390/ijerph16224321 (PMC6888482; doi:10.3390/ijerph16224321)
Supplement: Supplementary file 1 [file ijerph-16-04321-s001.pdf]

Supplemental Materials for Survey results of a pilot study on the effects of aircraft noise on sleep near Atlanta International Airport

The complete postal survey is given in *Questionnaire S1*
Demographic data for the sampled census tracts are given in Table S1
Directed acyclic graphs to determine minimal required adjustment for the regression models are given in Figures S1–S3
The number of respondents in each dichotomous outcome category is given in Tables S2–S4
Results of regression model statistical testing (*p*-values) are given in Table S5

Questionnaire S1 Complete postal survey

**Q1a**. During the **past month**, at what time have you usually gone to bed on weekdays or workdays?	______________________(HH:MM AM/PM)
**Q1b**. During the **past month**, at what time have you usually woken up on weekdays or workdays?	______________________(HH:MM AM/PM)
**Q1c**. During the **past month**, how much sleep did you usually get on weekdays or workdays?	______________________(Hours)

**Q2**. During the **past month**, how would you rate your sleep quality overall?			
Very Good ▼	Fairly Good ▼	Fairly Bad ▼	Very Bad ▼
☐	☐	☐	☐

Q3. For the following questions, select the response that best reflects how often the following occurred during the **past month.**

	Not during the past month ▼	Less than once a week ▼	Once or twice a week ▼	Three or more times a week ▼
**Q3a**. You had trouble sleeping because you cannot get to sleep within **30 minutes**?	☐	☐	☐	☐
**Q3b**. You had trouble sleeping because you wake up in the middle of the night or early morning?	☐	☐	☐	☐
**Q3c**. You have taken medicine (prescribed or “over the counter”) to help you sleep?	☐	☐	☐	☐
**Q3d**. You had trouble staying awake while driving, eating meals, or engaging in social activity?	☐	☐	☐	☐

**Q4**. How often in the **past month** have you done the following because of noise when trying to sleep at home?	Never 1 ▼	Rarely 2 ▼	Sometimes 3 ▼	Often 4 ▼	Always 5 ▼
**Q4a**. Wear earplugs or headphones	☐	☐	☐	☐	☐
**Q4b**. Use alcohol	☐	☐	☐	☐	☐
**Q4c**. Use medication	☐	☐	☐	☐	☐
**Q4d**. Turn on the TV	☐	☐	☐	☐	☐
**Q4e**. Turn on music	☐	☐	☐	☐	☐
**Q4f**. Close windows	☐	☐	☐	☐	☐
**Q4g**. Use a sound machine	☐	☐	☐	☐	☐
**Q4h**. Turn on a fan	☐	☐	☐	☐	☐

**Q5**. For the following statements respond how strongly you agree or disagree.	Strongly Disagree 1 ▼	2 ▼	3 ▼	4 ▼	5 ▼	Strongly Agree 6 ▼
**Q5a**. I am easily awakened by noise	☐	☐	☐	☐	☐	☐
**Q5b**. I get used to most noises without much difficulty	☐	☐	☐	☐	☐	☐
**Q5c**. I find it hard to relax in a place that is noisy	☐	☐	☐	☐	☐	☐
**Q5d**. I am good at concentrating no matter what is going on around me	☐	☐	☐	☐	☐	☐
**Q5e**. I get mad at people who make noise that keeps me from falling asleep or getting work done	☐	☐	☐	☐	☐	☐
**Q5f**. I am sensitive to noise	☐	☐	☐	☐	☐	☐

**Q6**. Thinking about the **last 12 months** or so, when you were here at home, how much was your sleep disturbed by noise from the following sources?	Not at all ▼	Slightly ▼	Moderately ▼	Very ▼	Extremely ▼
**Q6a**. Road Traffic	☐	☐	☐	☐	☐
**Q6b**. Trains	☐	☐	☐	☐	☐
**Q6c**. Aircraft	☐	☐	☐	☐	☐
**Q6d**. Industries/Factories	☐	☐	☐	☐	☐
**Q6e**. Construction	☐	☐	☐	☐	☐
**Q6f**. Neighbors	☐	☐	☐	☐	☐
**Q6g**. Air Conditioner	☐	☐	☐	☐	☐

Q7. Thinking about the **last 12 months** or so, when you are here at home, how much does noise from each of the following bother, disturb, or annoy you?

	Not at all	Slightly	Moderately	Very	Extremely
**Q7a**. Road Traffic	☐	☐	☐	☐	☐
**Q7b**. Trains	☐	☐	☐	☐	☐
**Q7c**. Aircraft	☐	☐	☐	☐	☐
**Q7d**. Industries/Factories	☐	☐	☐	☐	☐
**Q7e**. Construction	☐	☐	☐	☐	☐
**Q7f**. Neighbors	☐	☐	☐	☐	☐
**Q7g**. Air Conditioner	☐	☐	☐	☐	☐

Q8. In general, would you say your health is…?

Poor	Fair	Good	Very Good	Excellent
☐	☐	☐	☐	☐

Q9. Have you ever been diagnosed by a health professional with any of the following sleep disorders?

- ☐ Sleep Apnea
- ☐ Narcolepsy
- ☐ Restless Leg Syndrome
- ☐ Periodic Limb Movement Syndrome
- ☐ Insomnia
- ☐ None
- ☐ Other (please specify): ______________________________

Q10. Do you have any problems or difficulties with your sense of hearing? ☐ Yes ☐ No

**Q11a**. Have you ever been diagnosed by a health professional with the following conditions (mark all that apply)?	**Q11b**. If you have been diagnosed with a condition listed under Q11a, have you been treated for the condition in the **past month?**	
☐ Hypertension/High blood pressure	☐ Yes	☐ No
☐ Chronic headaches/Migraines	☐ Yes	☐ No
☐ Arrhythmia/Irregular heartbeat	☐ Yes	☐ No
☐ Heart disease	☐ Yes	☐ No
☐ Stomach ulcer	☐ Yes	☐ No
☐ Diabetes	☐ Yes	☐ No
☐ None of the above		

Q12. What is your marital status?

- ☐ Single
- ☐ Married
- ☐ Widowed
- ☐ Separated
- ☐ Divorced
- ☐ Domestic Partners

Q13. What was your total household income last year?

- ☐ < $25,000
- ☐ $25,000-$50,000
- ☐ $50,000-$75,000
- ☐ $75,000-$100,000
- ☐ $100,000-$150,000
- ☐ >$150,000
- ☐ Prefer Not to Answer

Q14. What is the highest level of education you have completed?

- ☐ Less than High School
- ☐ High School Graduate
- ☐ College Graduate or Higher

Q15. What is your current employment status?

- ☐ Working
- ☐ Unemployed
- ☐ Student
- ☐ Retired
- ☐ Homemaker

Q16. If currently employed, does your job require overnight shift work?

(Overnight shift work refers to work for at least 4 hours between 12 am midnight to 6 am in the morning)

☐ Yes ☐ No

Q17. Are you Hispanic or Latino? ☐ Yes ☐ No ☐ Prefer Not to Answer

Q18. What race do you consider yourself to be? (mark all that apply)

☐ American Indian or Alaska Native

☐ Native Hawaiian or Other Pacific Islander

☐ Asian

☐ White

☐ Black or African American

☐ Other (please specify):________________________

☐ Prefer Not to Answer

Q19. Has your current residence received any sound proofing treatment to reduce noise? ☐ Yes ☐ No

Q20. How long have you lived at your current residence?

☐ Less than 1 year

☐ 1-5 years

☐ 5-10 years

☐ More than 10 years

Q21. Do you have an air conditioner in your bedroom?

☐ No Unit

☐ Central Air Conditioner

☐ Window Unit

Q26. What is your height? __________feet__________inches

Q27. What is your weight? __________________________lbs

Q24. Gender: ☐ Male ☐ Female

Q25. Age: __________________________(years)

Q22. How many people (including yourself) reside in this household? __________________________

Q23. How many children in this household are under the age of 5? __________________________

Census tract-level demographic data

Table S1. Demographic characteristics of census tracts within each noise category.

Noise category	Direction re: ATL	Houses (n)	No college education (%)	Black or African American (%)	Mean household income ($)
≥55 dB	East	1949	59.5	55.1	33,624
≥55 dB	West	7305	50.8	90.7	26,737
50<55 dB	East	9464	59.7	59.6	31,126
50<55 dB	West	11,123	34.8	77.3	40,938
45<50 dB	East	14,489	46.3	83.6	46,964
45<50 dB	West	20,457	32.2	32.2	59,955
40>45 dB	East	53,391	41.9	77.4	50,249
40>45 dB	West	30,674	45.1	81.2	39,677
<40 dB	East	118,182	35.7	52.7	50,684
<40 dB	West	55,842	41.1	58.5	54,040
All	-	322,876	40.0	62.5	49,100

The total mean household income was calculated as (Σ(houses per tract × mean household income age per tract)) / total number of houses in all tracts. Similarly, the total mean percentages that had no college education or were Black or African American were calculated based on proportions and number of houses in each sampling region, e.g. (Σ(houses per tract × % per tract)) / total number of houses in all tracts.

Directed Acyclic Graphs (DAGs)

According to the DAGs, for all outcomes, to estimate the total effect of Lnight, the regression models should be minimally adjusted for age and household income.

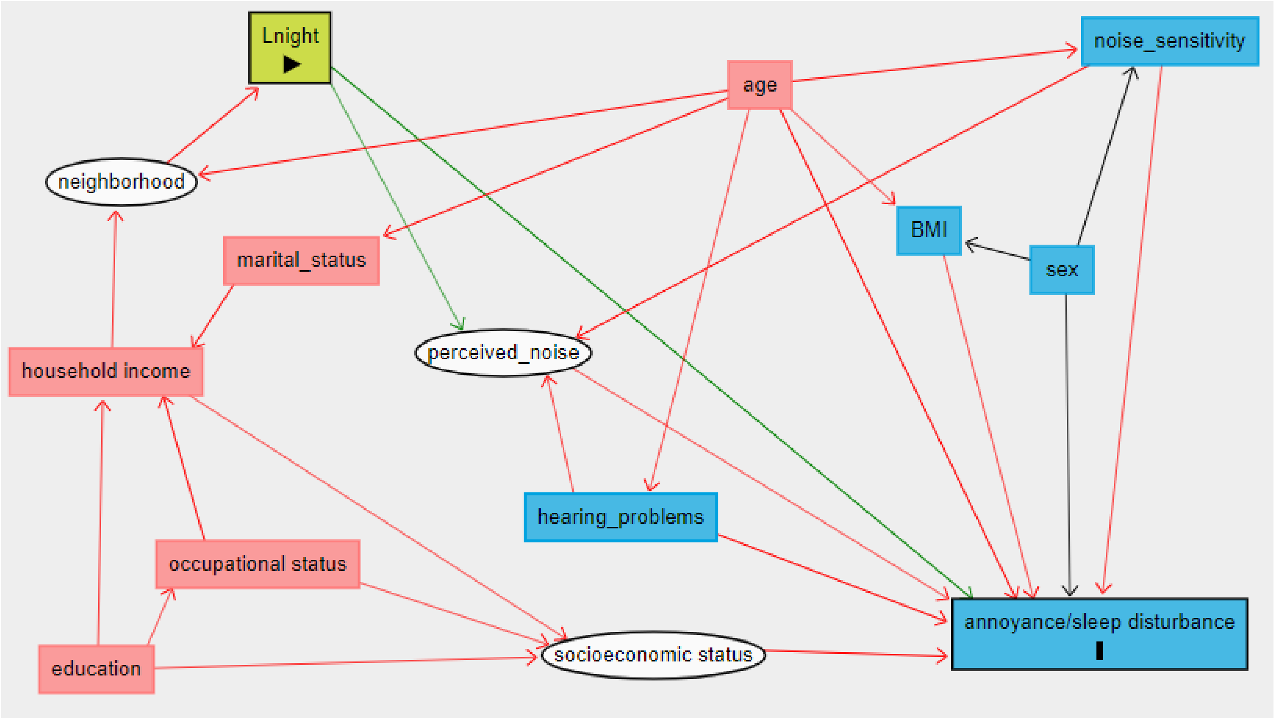

Figure S1. DAG for annoyance/sleep disturbance. To estimate the total effect of L_{night}, the model should be minimally adjusted for age and household income. Ovals indicate unmeasured variables.

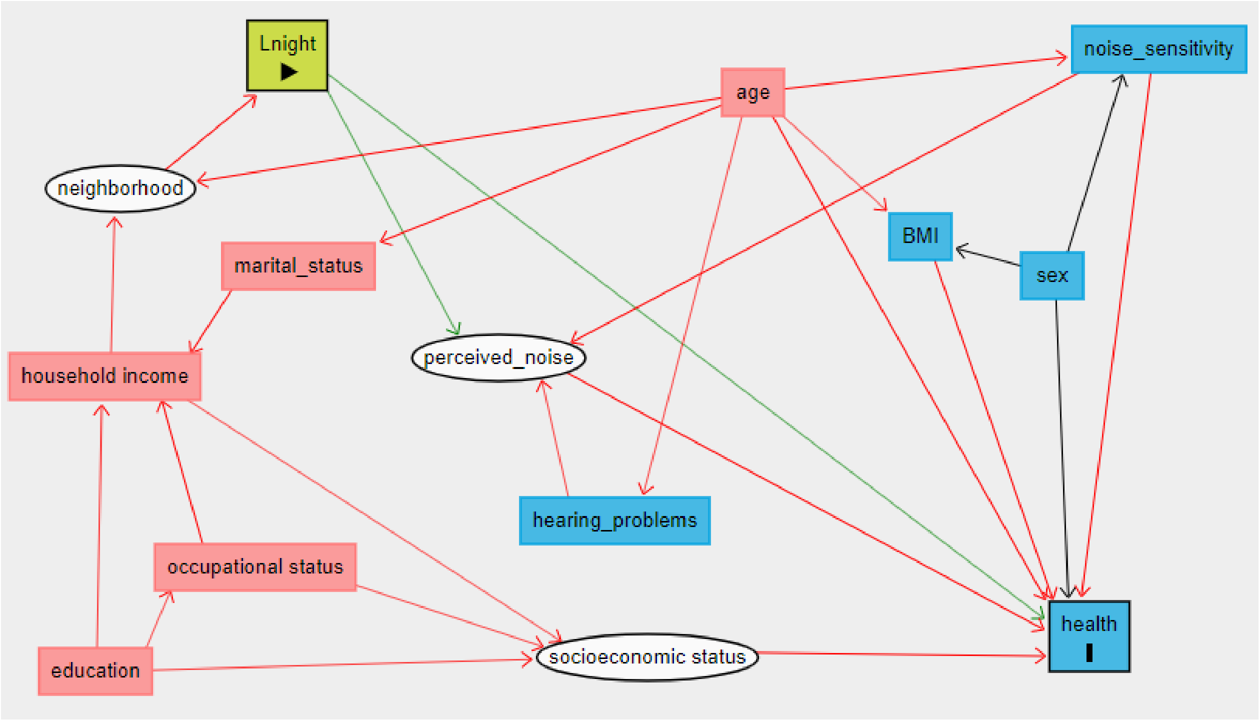

Figure S2. DAG for health outcomes. To estimate the total effect of L_{night}, the model should be minimally adjusted for age and household income. Ovals indicate unmeasured variables.

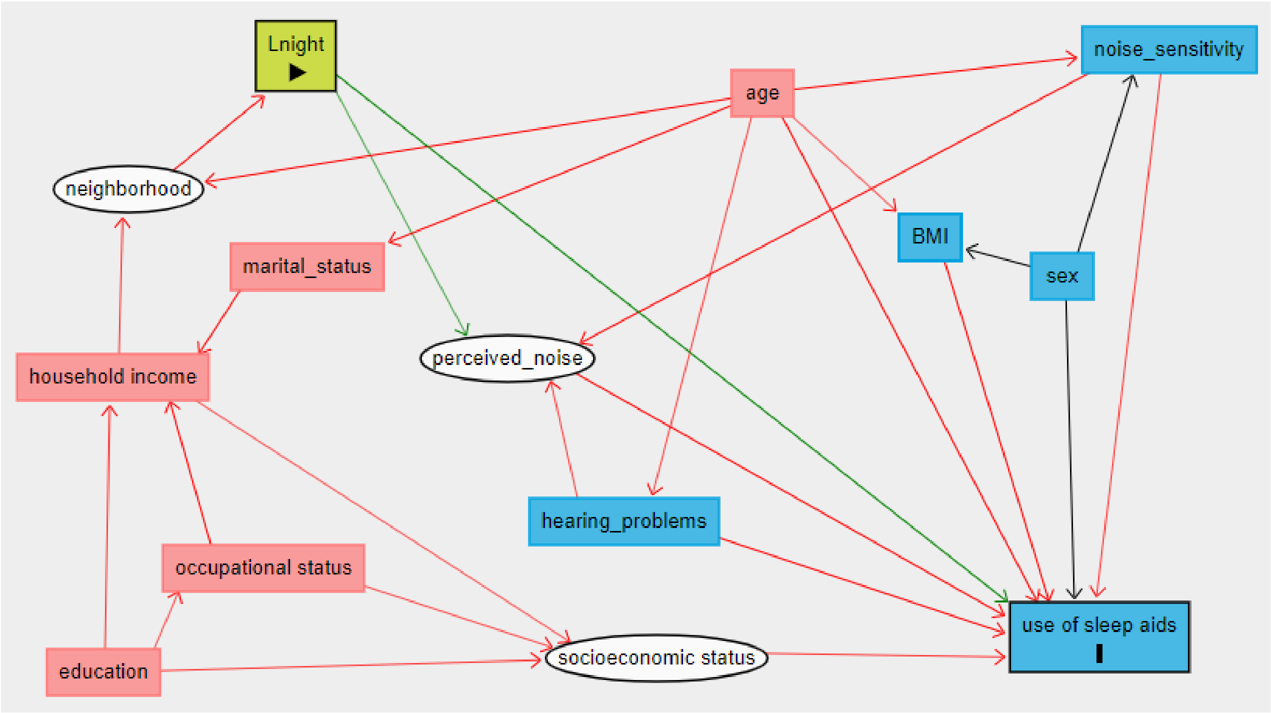

Figure S3. DAG for use of sleep aids. To estimate the total effect of L_{night}, the model should be minimally adjusted for age and household income. Ovals indicate unmeasured variables.

Prevalence of questionnaire outcome responses

These tables give the number of responses in each dichotomous response variable.

Table S2. Respondents (n) indicating being disturbed and annoyed by aircraft noise, fair or poor sleep quality, and trouble sleeping due to noise ≥1/week.

Response	Outcome Measure						
	**Sleep disturbance**	**Annoyance**	**Overall sleep quality**	**Trouble falling asleep**	**Trouble sleeping at night**	**Sleep medication**	**Trouble staying awake**
Disturbed/Annoyed/ Poor/≥1/week	83	78	86	146	176	45	41
Not disturbed/Not annoyed/ Poor/<1/week	180	188	176	116	86	211	217
Missing	5	2	6	6	6	12	10

Table S3. Respondents (n) indicating using sleep aids because of noise often or more frequently.

Response	Outcome							
	**Earplugs**	**Alcohol**	**Medication**	**TV**	**Music**	**Close windows**	**Sound machine**	**Fan**
Often or more	17	12	36	78	38	109	13	71
Sometimes or less	242	248	225	181	223	152	247	190
Missing	9	8	7	9	7	7	8	7

Table S4. Respondents (n) indicating poor or fair general health, diagnosis of any sleep disorder, and diagnosis of relevant health outcomes.

Response	Outcome Measure							
	**General health**	**Sleep disorder**	**Hypertension**	**Chronic headaches/ Migraine**	**Arrythmia**	**Heart disease**	**Stomach ulcer**	**Diabetes**
Poor/Yes	59	58	125	19	19	12	8	34
Good/no	207	210	143	249	249	256	260	234
Missing	2	0	0	0	0	0	0	0

Regression model p-values

Table S5. *p*-values for covariates included in the crude (L_{night} only) and fully adjusted regression model for each questionnaire outcome measure.

	Outcome measure	Model covariate									
		**L_{night} crude**	**L_{night} adjusted**	**BMI**	**Sex**	**Age**	**Hearing**	**Noise sensitivity**	**$50–100k**	**>$100k**	**Prefer not to answer**
Sleep	Sleep disturbance	<0.001	<0.001	0.022	0.889	0.929	0.498	0.001	0.065	0.022	0.326
	Annoyance	<0.001	<0.001	0.037	0.721	0.819	0.408	0.001	0.339	0.030	0.709
	Overall sleep quality	0.013	0.029	0.907	0.962	0.593	0.341	0.010	0.805	0.494	0.603
	Trouble falling asleep	0.002	0.003	0.748	0.453	0.177	0.029	0.001	0.862	0.272	0.191
	Trouble sleeping at night	0.019	0.040	0.864	0.074	0.146	0.044	<0.001	0.736	0.452	0.140
	Sleep medication	0.809	0.525	0.163	0.260	0.575	0.322	0.031	0.774	0.321	0.170
	Trouble staying awake	0.016	0.061	0.891	0.083	0.140	0.085	0.018	0.213	0.714	0.264
Coping	Earplugs	0.208	0.345	0.032	0.859	0.907	0.068	0.101	0.148	0.531	0.911
	Alcohol	0.028	0.046	0.868	0.869	0.161	0.589	0.187	0.796	0.926	0.447
	Medication	0.608	0.738	0.303	0.682	0.904	0.016	0.029	0.812	0.330	0.710
	TV	0.004	0.011	0.533	0.625	0.108	<0.001	0.022	0.452	0.889	0.058
	Music	0.004	0.020	0.160	0.675	0.000	0.001	0.855	0.514	0.332	0.989
	Close windows	0.007	0.006	0.074	0.603	0.146	0.889	0.055	0.961	0.282	0.066
	Sound machine	0.451	0.744	0.329	0.962	0.846	0.872	0.942	0.528	0.336	0.889
	Fan	0.212	0.496	0.116	0.407	0.007	0.065	0.020	0.538	0.996	0.811
Health	General health	0.004	0.052	0.001	0.398	0.565	0.049	0.469	0.529	0.056	0.536
	Sleep disorder	0.927	0.701	0.003	0.618	0.010	0.074	0.137	0.752	0.143	0.277
	Hypertension	0.858	0.990	<0.001	0.910	<0.001	0.606	0.673	0.705	0.168	0.821
	Chronic headaches/ migraine	0.192	0.383	0.613	0.254	0.225	0.752	0.550	0.760	0.357	0.469
	Arrythmia	0.525	0.689	0.840	0.813	0.002	0.202	0.330	0.690	0.943	0.708
	Heart disease	0.186	0.114	0.614	0.344	0.014	0.255	0.976	0.629	0.979	0.615
	Stomach ulcer	0.301	0.381	0.488	0.448	0.322	0.729	0.330	0.782	0.693	0.968
	Diabetes	0.448	0.119	0.001	0.662	<0.001	0.778	0.518	0.428	0.696	0.028
